# Immobilized Lentivirus Vector on Chondroitin Sulfate-Hyaluronate Acid-Silk Fibroin Hybrid Scaffold for Tissue-Engineered Ligament-Bone Junction

**DOI:** 10.1155/2014/816979

**Published:** 2014-06-12

**Authors:** Liguo Sun, Hongguo Li, Ling Qu, Rui Zhu, Xiangli Fan, Yingsen Xue, Zhenghong Xie, Hongbin Fan

**Affiliations:** ^1^Institute of Orthopedic Surgery, Xijing Hospital, The Fourth Military Medical University, Xi'an 710032, China; ^2^Department of Clinical Laboratory, Xijing Hospital, The Fourth Military Medical University, Xi'an 710032, China; ^3^College of Science, Air Force Engineering University, Xi'an 710051, China

## Abstract

The lack of a fibrocartilage layer between graft and bone remains the leading cause of graft failure after anterior cruciate ligament (ACL) reconstruction. The objective of this study was to develop a gene-modified silk cable-reinforced chondroitin sulfate-hyaluronate acid-silk fibroin (CHS) hybrid scaffold for reconstructing the fibrocartilage layer. The scaffold was fabricated by lyophilizing the CHS mixture with braided silk cables. The scanning electronic microscopy (SEM) showed that microporous CHS sponges were formed around silk cables. Each end of scaffold was modified with lentiviral-mediated transforming growth factor-**β**3 (TGF-**β**3) gene. The cells on scaffold were transfected by bonded lentivirus. In vitro culture demonstrated that mesenchymal stem cells (MSCs) on scaffolds proliferated vigorously and produced abundant collagen. The transcription levels of cartilage-specific genes also increased with culture time. After 2 weeks, the MSCs were distributed uniformly throughout scaffold. Deposited collagen was also found to increase. The chondral differentiation of MSCs was verified by expressions of collagen II and TGF-**β**3 genes in mRNA and protein level. Histology also confirmed the production of cartilage extracellular matrix (ECM) components. The results demonstrated that gene-modified silk cable-reinforced CHS scaffold was capable of supporting cell proliferation and differentiation to reconstruct the cartilage layer of interface.

## 1. Introduction

The rupture of the anterior cruciate ligament (ACL) is one of the most common injuries of the knee with an incidence of 1 in 3000 [1], which can result in severe limitations in mobility, pain, and inability to participate in sports and exercise. Due to the limited capacity to regenerate, ACL heals poorly in response to the repair by suturing the injured tissue back together. So the grafts are required for ACL reconstruction [2]. Currently, there are approximately 125,000 ACL reconstruction surgeries performed worldwide each year, most using biological grafts (autografts and allografts). However, there are still drawbacks including the risk of disease transmission, lack of appropriate donors, immune response, and high costs [3, 4]. Recently tissue engineering has emerged as a promising strategy for the regeneration of injured ligament with similar biomechanical and biochemical properties [4, 5]. The scaffold plays an important role in constructing the tissue-engineered ligament by providing appropriate mechanical integrity and biochemical stimulation.

The integration between soft graft and hard bone-tunnel is particularly critical for biological fixation. The native structure of ACL-bone insertion has four distinct yet continuous regions of ligament, noncalcified fibrocartilage, calcified fibrocartilage, and bone. Interface tissue engineering is a new strategy aiming at regeneration of interface and ultimately enabling the biological fixation of soft grafts in bone-tunnel [6]. The intricate multitissue organization of insertion indicates that interface scaffold design must consider the need to regenerate more than one type of tissue, as well as exercising spatial control over the respective cell populations indigenous to different interface regions. Initial attempts to improve ligament graft to bone fixation focused on augmenting the surgical graft with a material that could encourage bone tissue ingrowth. In previous studies, the scaffold modified by calcium phosphate or tricalcium phosphate cement was found to enhance healing and promote integration [7–9]. Additional approaches to improve osteointegration included the addition of periosteum grafts to the region of graft that interacted with the bone and growth factors such as rhBMP-2 [10–14]. Although these methods have improved osteointegration between graft and bone-tunnel, the efforts do not result in regeneration of fibrocartilage layer of interface. Moreover, single-phase scaffold does not fully mimic the complexity of natural interface. The ideal biomimetic scaffold should be designed to recapitulate the inherent complexity of multilayered ligament-to-bone interface. It might direct the growth of the multitissue and overcome shortcomings mentioned above. This multilayer interface scaffold was reported to be loaded with different cells (fibroblast, chondrocyte, and osteoblast) for reconstructing insertion. But it is difficult to conduct due to complicated process.

In recent years, silk has been increasingly studied as the scaffold for ligament tissue engineering due to its biocompatibility, slow degradability, and remarkable mechanical capability [15, 16]. In our previous work, the knitted scaffold was demonstrated to possess good mechanical strength and internal connections. It could facilitate nutrients' transmission, tissue infiltration, and matrix deposition. After implantation the scaffold could successfully regenerate ACL in small and large animal models. However, the typical four layers of insertion were not observed, which might decrease the stability of graft [17]. Growth factors have long been delivered to cells to promote cell growth, proliferation, and differentiation. Transforming growth factor-*β*3 (TGF-*β*3) was substantiated to help to maintain a round or polygonal shape of chondrocytes and stimulate total collagen synthesis, which is crucial in maintaining the cartilage functions [18, 19]. Recombinant TGF-*β*3 protein has also been delivered to mesenchymal stem cells (MSCs) for in vitro chondrogenesis. In our previous work, the immobilized TGF-*β*3 on scaffold efficiently induced the chondrogenic differentiation of MSCs [20]. In this study, the bone-tunnel part of scaffold was modified with lentivirus carrying TGF-*β*3 gene. We hypothesize that MSCs transfected by lentivirus can regenerate cartilage-like tissue between ligament and bone-tunnel to resemble cartilage layer of native insertion.

In order to prove this hypothesis, the study was designed (1) to prepare a silk cable-reinforced chondroitin sulfate-hyaluronate acid-silk fibroin (CHS) scaffold modified with lentiviral-mediated TGF-*β*3 gene, (2) to observe cell proliferation and collagen production in modified area of scaffold, and (3) to examine expressions of cartilage related genes in mRNA and protein level with real-time quantitative RT-PCR, western bolt, and histological stains. The purpose of current study is to find whether the cartilage layer of ligament-bone junction can be reconstructed in vitro using gene-modified scaffold and MSCs.

## 2. Materials and Methods

### 2.1. Scaffold Fabrication

**Figure 1:**
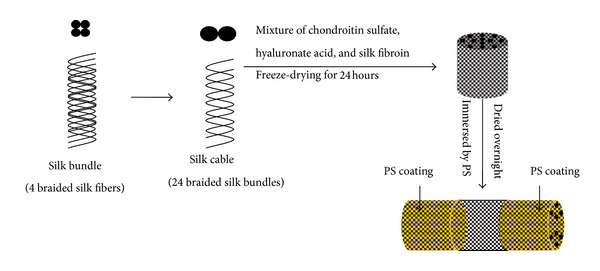
Schematic outline of scaffold fabrication procedure.

Four sericin-free silk fibers were braided into one bundle and then two bundles were braided into one cable. Twelve cables placed in parallel were fixed across a customized polypropylene cylinder mold (diameter: 6 millimeters; length: 60 millimeters). The raw Bombyx Mori silk fibers were immersed in 0.02 M NaHCO_3_ solution at 90°C for 1 hour to remove sericin. The fibers were rinsed for 20 minutes 3 times, squeezed out excess water, and allowed to dry overnight. To prepare silk fibroin (SF) solution, the sericin-free silk fibers were added to 9.3 M lithium bromide (BrLi) solution on top of silk fibers and incubated at 60°C for 4 hours [21]. After dialysis with SnakeSkin (Thermo Scientific Co., 3500 MWCO, USA), the final concentration of SF was 2.0 wt%. The 0.1 g of chondroitin sulfate sodium (Sigma Co., St. Louis) was mixed with 5 mg of hyaluronate (C. P. Freda Pharmacy Co., Shandong, China) and 12 mL of 2.0 wt% SF solution and then poured into the cylinder mold containing the 12 silk cables. Before lyophilization the cylinder mold was kept at −80°C for 1 hour and then put into lyophilizer (Christ Alpha 1-2 LD, Germany) for 24 hours. The freeze-dried scaffold was immersed in 90% methanol solution for 30 minutes to induce *β*-sheet structural transition and then washed several times to remove the residual chemicals. Thereafter, the end of scaffold (length 20 millimeters) was immersed in phosphatidylserine (PS) chloroform solutions for 5 minutes. Finally, the scaffold was dried overnight in hood. The process was schematically depicted in Figure 1.

### 2.2. Scaffold Characterization

The structures and mechanical properties of scaffold were characterized. Mercury porosimetry (Pascal 140, GA) was used to assess the pore size, porosity, and total surface area of scaffold (*n* = 4). Scanning electronic microscopy (SEM) (HITACHI S-4800, Japan) was used to observe the morphology of micropores. To measure hydrophilicity, the dried scaffolds (*n* = 6) were hydrated in phosphate buffered saline (PBS) after being weighed. Then the swollen scaffolds were weighed again and the degree of swelling was calculated as the ratio of the weight of PBS absorbed by scaffolds normalized to the initial dry weight [22]. Mechanical test was performed using Shimadzu mechanical testing system (AGS-10kNG, Shimadzu Inc., Japan) with a maximum loading capacity of 500 N. After being hydrated in PBS, the 12 cable-reinforced CHS hybrid scaffolds (*n* = 6) were performed with a gauge length of 20 millimeters at a loading speed of 2 mm/min. The maximum tensile load (max-load), maximum tensile distance (max-disp), break tensile load (break-load), and break tensile distance (break-disp) were determined.

### 2.3. Isolation and Expansion of MSCs

MSCs were generated from bone marrow aspirates of rabbits (12 weeks old, 2.5–3.0 kg). According to previous methods [20], mononuclear cells were separated by centrifugation in a Ficoll-Paque gradient (Sigma Co., St. Louis) and suspended in 20 mL of Dulbecco's modified eagle medium (DMEM) supplemented with 15% fetal bovine serum (FBS) (HyClone Logan, Utah). Cultures were incubated at 37°C and 5% carbon dioxide for 72 hours; then the nonadherent cells were removed by changing medium. When reaching 70–80% confluence, adherent cells were detached from the flask using 0.25% trypsin and subcultured. A homogenous MSCs' population was obtained after 2 weeks of culture and cells of passage 3 were harvested for further use. The adipogenic, osteogenic, and chondrogenic differentiations of cells were tested to ensure the multilineage potential.

### 2.4. Cell Adhesion, Metabolism, Viability, and Transfection

Cell adhesion to scaffold was examined as reported [23]. The hybrid scaffolds were sliced into circular disc (diameter: 6 mm; length: 4 mm), sterilized by brief treatment with 75% ethanol. The lentivirus vector (1 × 10^8^ copies/mL) with a promoter encoding both TGF-*β*3 and enhanced green fluorescent protein (eGFP) was prepared by GeneCopoeia Co.

Then the scaffold was incubated with transfection medium (40 × 10^6^ of virus in 1 mL of DMEM) at 37°C and 5% CO_2_ 4 hours for immobilization of lentivirus vector. The scaffold was placed into a polypropylene tube (length: 25 millimeters; inner diameter: 6.0 millimeters), loaded with 1 × 10^6^ MSCs (passage 3), and incubated. At different time points (i.e., 0, 0.5, 1, 2, 4, and 8 hours) the MSCs/scaffolds (*n* = 6 for each time point) were gently rinsed with PBS. The detached cells collected from rinsing solution and medium were counted using light microscopy.

The metabolism of MSCs on scaffold was evaluated at 1, 3, 5, 7, 9, 11, 13, and 15 days by alamar blue assay following the vendor's instructions (ABD Serotec, Oxford, USA). The alamar blue assay is a useful method for cell monitoring on 3D porous scaffold [17]. A brief description was as follows: the MSCs/scaffold (*n* = 6 for each culture period) was incubated in culture medium supplemented with 10% (v/v) alamar blue fluorescent dye for 2 hours. Then 100 *μ*L of medium was extracted from each sample and measured at 570/600 nm in a microplate reader (Sunnyvale, CA). The culture medium supplemented with 10% alamar blue was used as a negative control.

The viability of MSCs seeded on scaffolds (*n* = 4) was examined at culture periods of 1 and 2 weeks using live/dead cells assay. The assay was based on the combination of the fluorescein diacetate (FDA), which stains living cells green, and propidium iodide (PI), which stains dead cells red. Briefly, the MSCs/scaffolds were thoroughly rinsed with PBS in a six-well plate. Then 2 mL of PBS supplemented with 2 *μ*L of PI (2 mg/mL) and 6 *μ*L of FDA (5 mg/mL) was added into each well. The system was allowed to incubate at room temperature for 10 minutes. Then, each sample was washed with PBS twice and observed with confocal microscopy (Olympus Fluo View FV-1000, Japan).

The transfection of MSCs was examined after 1 and 2 weeks using fluorescence microscope (Leica DMI6000B Inverted Microscope, Germany). The MSCs/scaffolds (*n* = 4 for each time point) were gently rinsed with 1 mL of PBS, added to 3 mL of trypsin-EDTA solution (Beyotime Co., China), and shaken for 20 minutes. The detached cells were collected, resuspended in DMEM, and transferred into flask for cells adhesion. After 4 hours of culture, the transfected cells which expressed the enhanced green fluorescent protein (eGFP) were observed with fluorescence microscope.

### 2.5. Collagen Production

One million MSCs were loaded onto scaffold and cultured in vitro for periods of 1 and 2 weeks. The collagen deposited on scaffold was then quantified using sircol collagen dye binding assay kit (Biocolor Ltd., Newtownabbey, Ireland). The dye reagent specifically binds to the [Gly-X-Y]n helical structure of collagen but not to unwound triple helix or random chains of gelatin. Briefly, the samples (*n* = 6) were incubated with 500 *μ*L of pepsin solution (0.25 mg/mL) and shaken for 2 hours. Then 1 mL of dye reagent was added to 300 *μ*L of soluble collagen and mixed for 30 minutes at room temperature. The pellet of dyed collagen was precipitated by centrifugation for 5 minutes and then dissolved with 1 mL of releasing reagent. The absorbance was measured at 540 nm. The standard curve was set up on the basis of collagen standard provided by the vendor. The collagen produced was presented as amount of collagen per scaffold.

### 2.6. Histological Assessment

MSCs seeded on the scaffolds (*n* = 6) were cultured and harvested at the end of 1 and 2 weeks. The samples were washed with PBS and fixed in 10% neutral buffered formalin. Thereafter, they were dehydrated through a series of graded alcohols and embedded in paraffin. Sections of 5 *μ*m thickness were cut and collected on slides. For immunohistochemistry stains, the slides were incubated with collagen II (Sigma Co.) and TGF-*β*3 (Sigma Co.) antibodies. Then detection was applied using streptavidin-biotin immunoenzymatic antigen detection system (UltraVision Detection System, LabVision, USA). The results were assessed by three individuals who were blinded to the treatment.

### 2.7. Real-Time Quantitative RT-PCR Analysis

Total RNA was extracted from MSCs/scaffold (*n* = 6) constructs at the end of 1 and 2 weeks using the RNeasy mini kit (Qiagen, Valencia, CA). RNA concentration was determined by using NanoDrop (NanoDrop Technologies, Wilmington, DE) and 200 ng of RNA was used to synthesize cDNA with Iscript cDNA synthesis kit (Biorad Laboratories, Hercules, CA). Quantitative real-time PCR measurement was carried out using the Stratagene Mx3000P system (Stratagene, La Jolla, CA, USA). QuantiTect SYBR Green PCR kit (Qiagen, Valencia, CA, USA) was used to quantify the transcription level of collagen II and TGF-*β*3. Sequences of all the primers for real-time PCR were as follows (5′–3′): TGF-*β*3, TGG CTG TTG AGA AGA GAG TCC, TGC TTC AGG GTT CAG AGT GTT; collagen II, AAC ACT GCC AAC GTC CAG AT, CTG CAG CAC GGT ATA GGT GA. cDNA of 1 mL from each sample was mixed with 10 *μ*L of QuantiTect SYBR Green PCR master mix, 0.25 *μ*L of primer, and 8.5 *μ*L of RNase-free water. The total reaction volume was 20 *μ*L. Real-time PCR reactions were performed at 95°C for 15 minutes, followed by 40 cycles of amplification consisting of denaturation step at 95°C for 15 seconds and extension step at 60°C for 1 minute. The transcription level normalized to GAPDH was then calculated using the 2ΔCt formula with reference to the undifferentiated MSCs.

### 2.8. Western Blot Analysis

Proteins were extracted from MSCs/scaffold (*n* = 4) at the end of 1 and 2 weeks with pepsin (200 *μ*g/mL in 0.08 M acetic acid, Sigma) for 72 hours at 4°C. The pepsin was subsequently inactivated with 1 M NaOH. The extract was concentrated using a Nanosep 30 centrifugal filter (30,000 Mw cutoff, Pall Life Sciences, USA). Samples were then separated by electrophoresis in NuPAGE Novex Tris-acetate mini gels (Invitrogen, USA) and electrophoretically transferred to a supported nitrocellulose membrane (Biorad Laboratories). The membranes were tested using western blot kit (Invitrogen, USA) according to the manufacturer's instructions. A brief description was as follows: the membranes were blocked with buffer for 1 hour and incubated overnight at 4°C with monoclonal antibodies against collagen II and TGF-*β*3 diluted to 1 : 500 in blocking buffer. The membranes were then washed five times with washing buffer and incubated for 30 minutes with secondary antibodies diluted to 1 : 200 in blocking buffer. The membranes were rinsed with washing buffer again and incubated with ECL working solution for 5 minutes. The signal was detected using VersaDoc Imaging System (Biorad Laboratories) and relative intensities of positive bands were compared between groups.

### 2.9. Statistical Analysis

The mean and standard deviation were used to describe the data. The data analysis was performed using SPSS Statistics 20.0 statistical software package. A statistical analysis of quantitative results was carried out with the unpaired Student's* t*-test and one-way analysis of variance (ANOVA). The statistical significance level was set at 0.05.

## 3. Results

### 3.1. Characterization of Scaffold

**Figure 2:**
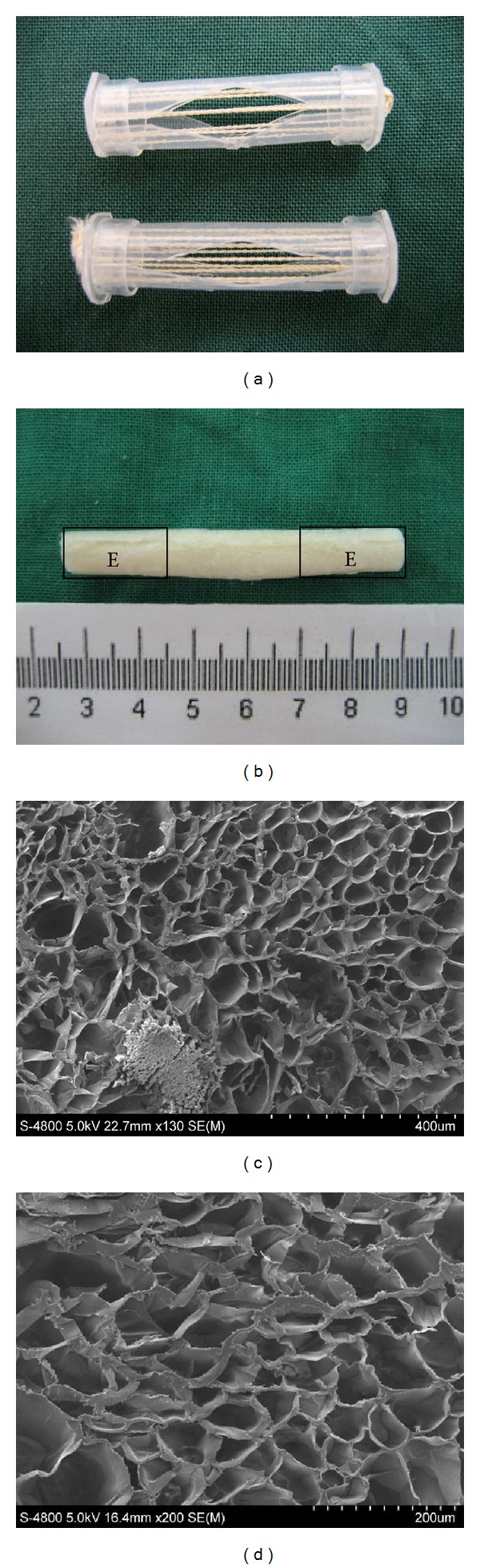
(a) Customized polypropylene cylinder mold containing 12 silk cables. (b) Gross morphology of scaffold and the zone encoded E was coated with PS. (c) SEM image of microporous structure of scaffold (×85) and hybrid microsponge around silk cable. (d) The scaffold displayed a highly porous microstructure with a high degree of interconnectivity (SEM × 150).

**Figure 3:**
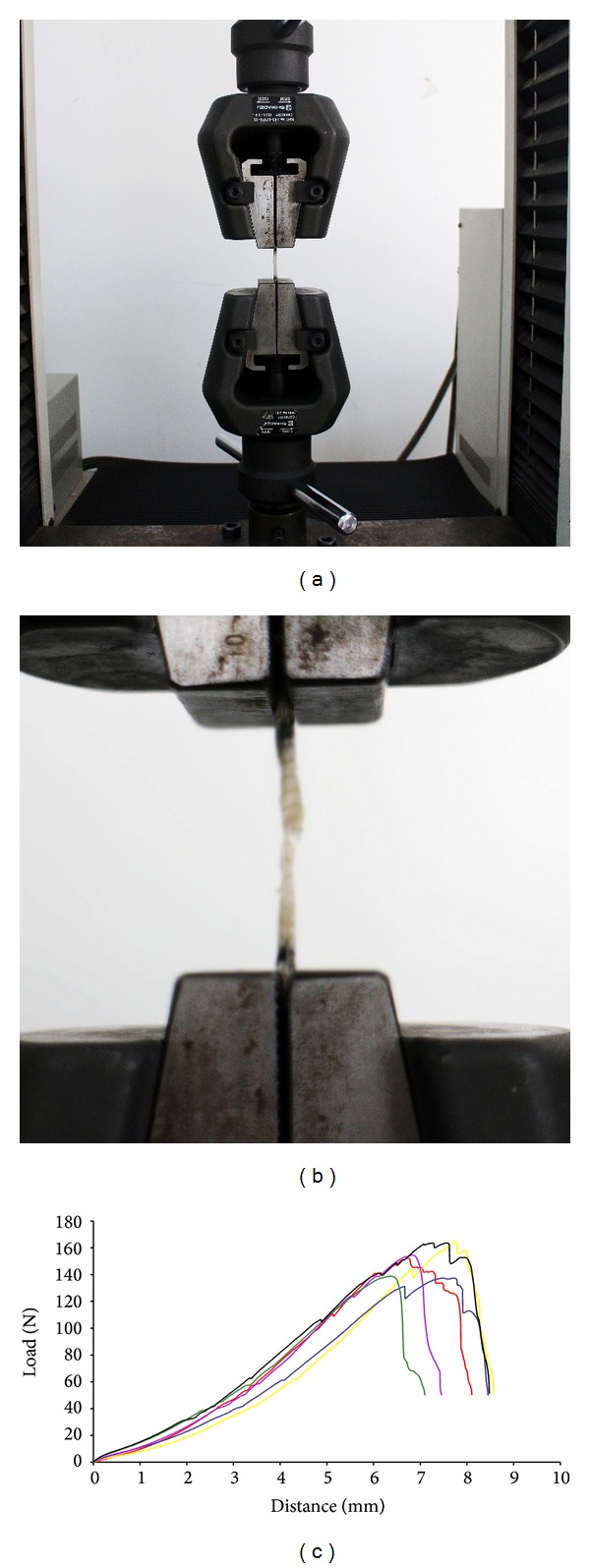
Mechanical properties of regenerated ligaments. (a) The scaffold was firmly fixed on machine to perform mechanical test. (b) The scaffold broke in the midsubstance during mechanical test. (c) The load-deformation curves of scaffold.

Twelve cables placed in parallel were fixed across a customized polypropylene cylinder mold (Figure 2(a)). The cable-reinforced CHS hybrid scaffold exhibited an elastic texture and porous morphology. Each end of scaffold (length 20 millimeters) in the E areas (Figure 2(b)) was immersed in PS chloroform solutions. The porosity of the microporous sponge was found to be 63.4 ± 4.2% and the average pore diameter was 172.3 ± 52.6 *μ*m. The surface area of the hybrid scaffold was 2.1 ± 0.3 m^2^/g. The SEM images also indicated that the pore size ranged from 80 to 230 *μ*m (Figures 2(c) and 2(d)). The swelling ratio of the scaffold was measured to be 635.7 ± 48.3%.  Figure 3 showed the summary of mechanical properties of the scaffold. The max-load was 151.9 ± 11.7 N and the max-disp was 7.1 ± 0.6 mm. The break-load was 51.3 ± 1.0 N and the break-disp was 8.0 ± 0.6 mm.

### 3.2. Cell Adhesion, Metabolism, Viability, and Transfection

**Figure 4:**
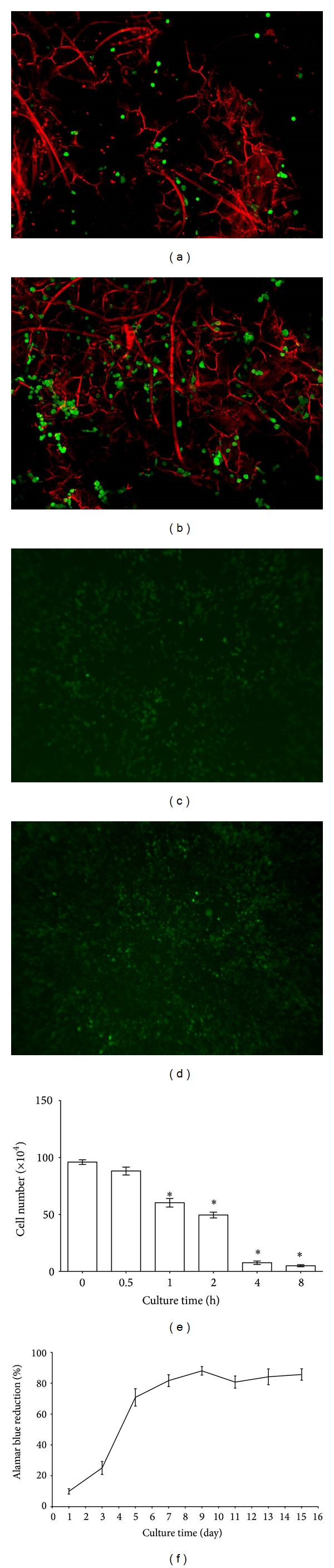
(a, b) The viability of MSCs seeded on scaffold evaluated by confocal microscope at 1-week (a) and 2-week (b) culture period (FDA/PI staining; green represents live cells; red represents dead cells). (c, d) The transfection of MSCs observed by fluorescence microscope at 1-week (c) and 2-week (d) culture period (×40). (e) Nonadherent cells count at 0, 0.5, 1, 2, 4, and 8 h time points after cell seeding (mean ± SD, *n* = 6, *P < 0.05). (f) The value of alamar blue assay on days 1, 3, 5, 7, 9, 11, 13, and 15 after cell seeding (mean ± SD, *n* = 6).

The results indicated that the number of nonadherent cells decreased proportionately with increasing culture time. The number of nonadherent cells at the 4 h group was 7.7 ± 1.4 × 10^4^. This was significantly lower than cell number of 0 h group (*P* < 0.05). Although the cell number continued to decrease after 4 hours, there was no significant difference between 4 h and 8 h groups. Therefore, 4 h incubation was deemed sufficient for MSCs to attach onto hybrid scaffold (Figure 4(e)).

The metabolism of MSCs seeded on the hybrid scaffold increased rapidly with culture time at early stage. The value of alamar blue test increased from 9.9 ± 1.6% to 70.8 ± 5.1% after 5 days of culture. Subsequently it increased gradually and reached a plateau. The value after 15 days was 85.7 ± 3.4% (Figure 4(f)). Viability of MSCs seeded on scaffold was evaluated by confocal microscope. Based on live/dead assay, no significant cell death was observed (Figures 4(a) and 4(b)). The transfected cells expressing eGFP were observed in the interconnective pores with fluorescence microscope. The number of transfected cells at 2-week time points significantly increased compared to that of 1-week ones (Figures 4(c) and 4(d)).

### 3.3. Collagen Production

**Figure 5:**
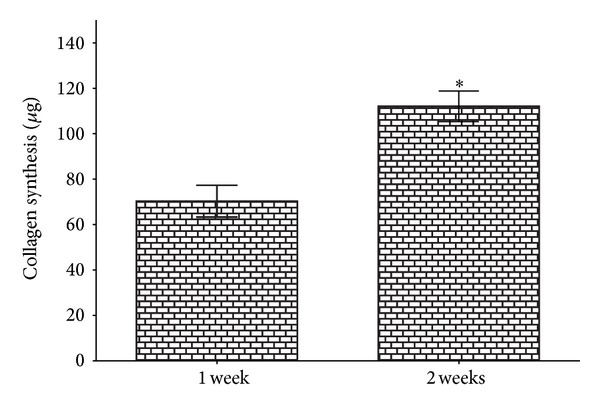
Amount of collagen produced by MSCs seeded on hybrid scaffold at 1-week and 2-week culture periods (mean ± SD, *n* = 6, *P < 0.05).

Collagen deposition on hybrid scaffold was found to increase proportionately with culture time. The MSCs produced an average of 79.2 ± 4.5 and 112.1 ± 6.3 *μ*g collagen after culture periods of 1 and 2 weeks, respectively. The difference between these two groups was found to be significant (*P* < 0.05) (Figure 5).

### 3.4. Histological Analysis

**Figure 6:**
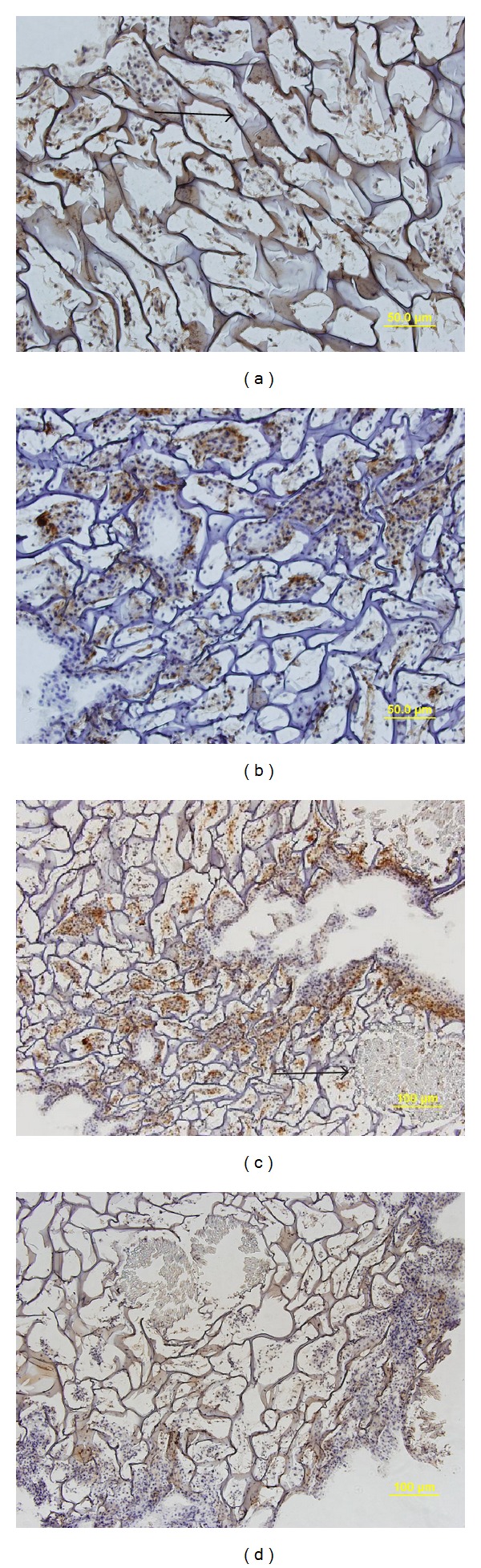
Histological evaluation of MSCs on hybrid scaffold at 1-week (a, b) (×200) and 2-week (c, d) (×100) culture period by immunohistochemistry staining specific for TFG-*β*3 (a, c) and collagen II (b, d). The MSCs proliferated robustly and produced abundant extracellular matrix. The thick arrow indicates silk cable and the thin arrow indicates the hybrid scaffold CHS microsponges.

Histological examination revealed that microporous structure of the hybrid scaffold was well preserved and few micropore walls collapsed. Thus, the exchange of nutrients between scaffold and environment was ensured. The MSCs proliferated robustly along the wall of micropores and exhibited fibroblast morphology with elongated nucleus. The 2-week group showed higher cell density and more ECM production when compared with 1-week group. Immunohistochemistry staining for collagen II and TGF-*β*3 was found to be positive after 1 week. ECM formation was observed to increase with culture time. The staining of collagen II and TGF-*β*3 was more intense at 2 weeks when compared with that at 1 week (Figure 6).

### 3.5. Transcription Level of Cartilage-Specific Genes

**Figure 7:**
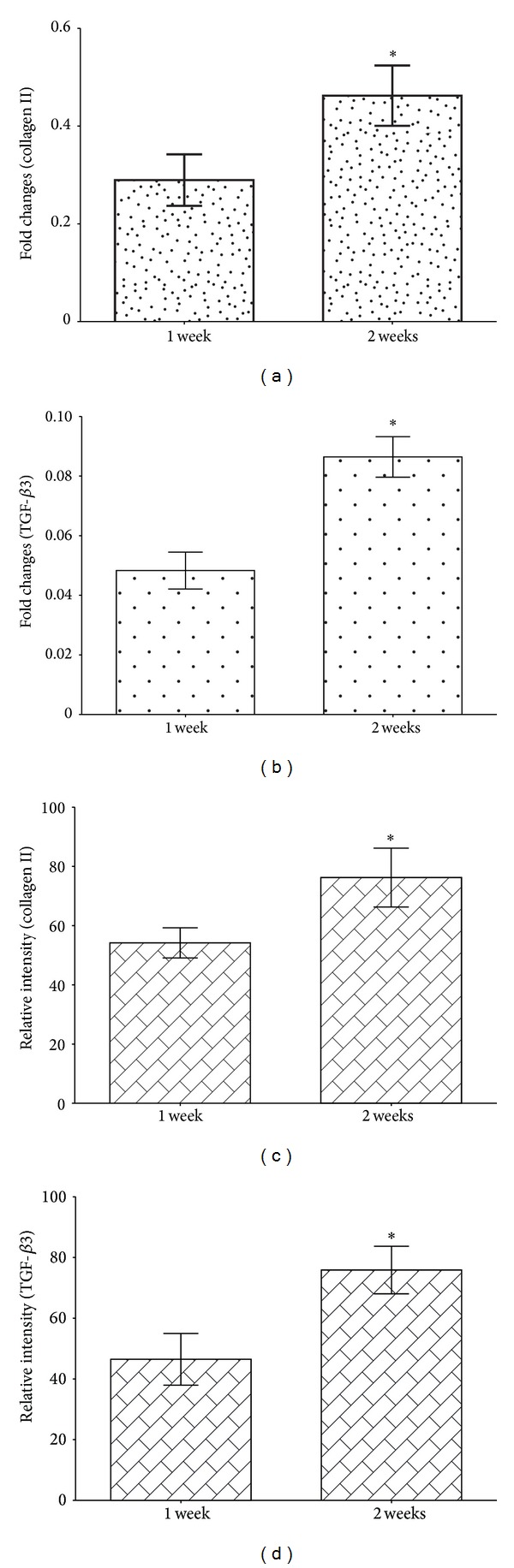
The transcription levels of collagen type II gene (a) and TGF-*β*3 gene (b) in RT-PCR assay at 1-week and 2-week time points; the relative intensities of positive staining band for collagen II (c) and TGF-*β*3 (d) in western blot assay at 1-week and 2-week time points (*P < 0.05).

MSCs cultured on hybrid scaffold were harvested to assess their gene transcription level using real-time quantitative RT-PCR. The transcription levels of collagen II gene were 0.290 ± 0.046-fold and 0.462 ± 0.053-fold at 1 and 2 weeks (*P* < 0.05), respectively. The results showed that TGF-*β*3 gene transcription was 0.048 ± 0.005-fold and 0.086 ± 0.006-fold at at 1-week and 2-week time points. The difference at these two time points was found to be significant (*P* < 0.05) (Figures 7(a) and 7(b)). The results suggested that the hybrid scaffold could support the differentiation of MSCs towards chondrocytes.

### 3.6. Western Blot Analysis

The protein expressions of TGF-*β*3 and collagen II were compared between groups after 1 week and 2 weeks of culture. Collagen II and TGF-*β*3 were expressed more prominently in 2-week groups in comparison with 1-week groups. The relative intensities of positive staining band for collagen II were 54.2 ± 4.4% and 76.2 ± 8.6% after 1 week and 2 weeks, respectively. There was a significant difference (*P* < 0.05). The relative intensities of positive staining band for TGF-*β*3 were 46.5 ± 7.4% and 75.9 ± 6.8% after 1 week and 2 weeks, respectively. There was a significant difference (*P* < 0.05) (Figures 7(c) and 7(d)).

## 4. Discussion

This study demonstrated that the lentivirus vector could be immobilized by PS on scaffold, which transfected MSCs continuously. The transfected cells could differentiate into chondrocyte-like cell. This tissue-engineered cartilage layer mimicked the chondral part of natural ligament-bone junction. It might reduce the stress concentration between hard bone and elastic ligament. The results provide potential application of gene-modified scaffold for constructing the cartilage-zone of ligament-bone junction.

The adherence between vector and material was achieved by the binding between lentivirus and PS coating, a component of the plasma membrane. Shin et al. reported that PS could be incorporated into PLG microspheres, which was subsequently combined with scaffold. Large numbers of transduced cells and increased gene expression were observed on the gene-modified scaffold [24]. In this study, lentiviral vector was immobilized on scaffold by incorporation with PS.

The integration of gene therapy into tissue engineering to control differentiation and direct tissue formation is not a new concept; however, successful delivery of nucleic acids into primary cells, progenitor cells, and stem cells has proven exceptionally challenging. The gene delivery methods include viral and nonviral methods. The usually used physical, nonviral methods are microinjection, ballistic gene delivery, electroporation, sonoporation, laser irradiation, magnetofection, and electric field-induced molecular vibration. However, the clinical application of nonviral methods is still restricted by some limitations including low transfection efficiency and poor transgene expression. Viral vectors are generally highly effective at delivering nucleic acids to a variety of cell populations. The commonly used vectors mainly include adenovirus, adeno-associated virus (AAV), and retroviruses. Adenovirus and AAV are hardly integrated into target cell genome. Lentivirus belongs to a genus of retrovirus; it can effectively integrate exogenous gene into the host chromosome, so as to achieve the persistence [25]. It is very important that the lentivirus transfection system does not produce cell damage and immune response [26].

A significant role of PS is to increase the amount of virus that associates with the material. Thus a higher dosage of vector can be delivered locally. For scaffold modification, we once demonstrated an increase in lentivirus activity when compared with virus alone (unpublished data). In this study, we found very high transduction efficiency of MSCs with an MOI (multiplicity of infection) of 40 on the scaffold immobilized by PS. At this MOI, over 90% of the cells were identified to be transfected by fluorescence microscope. Despite these high transfection efficiencies, there was no evidence of cell death at this MOI. Lentiviral vectors can transfect both replicating and nonreplicating cells. It can be incorporated into the host genome, thereby theoretically offering prolonged protein production. Although long-term protein production may enhance cartilage repair, there are also concerns about the formation of potential oncogenic effects on surrounding cells.

Several methods have been used to immobilize virus on scaffold. Although these approaches have been effective, vector biotinylation can influence its activity [27, 28]. PS has a specific interaction with the VSV-G protein, which is influenced in part by electrostatic interactions. PS is a relatively low molecular weight lipid that is soluble in organic solvents, which is relatively easy compared with the immobilization of antibodies or avidin for virus binding.

In general, a scaffold with a minimal pore size of 150 *μ*m is suggested for bone tissue engineering. For soft tissue engineering, the pore size of the scaffold usually ranges from 150 to 250 *μ*m. The newly formed ECM on the surface would further prevent the cells from infiltrating into the scaffold [29]. It has been estimated that the ligament typically bears peak loads of about 169 N during normal ambulation, with a threefold increase from 400 N to 500 N during strenuous athletic activity [30]. In this study the average pore size of the hybrid scaffold was 172.3 ± 52.6 *μ*m, which was suitable for ligament tissue engineering. The enlarged pores and high porosity of scaffold facilitated tissue ingrowth, as demonstrated by histology examination. The hybrid scaffold has a microporous structure with interconnecting pores, consequentially enlarging the surface area for better cell adhesion. However, microporous structure can result in poor mechanical properties. To overcome this drawback, braided silk cables were introduced to increase tensile strength. In this study, the hybrid scaffold was designed to have an average max-load of 151.9 ± 11.7 N. This would meet the mechanical requirement of ACL for activities of daily living. Because the max-load transmittable through the hybrid scaffold is proportional to the number of silk cables, the tensile strength of scaffold can be easily adjusted by changing the number of supporting silk cables.

An important aspect of ECM is its ability to store water, which is necessary to support various cell activities and nutrients exchange. The weight swelling ratio of silk cable-reinforced CHS scaffold was 635.7 ± 48.3%. This is about four times higher than that of sericin-free silk fiber (137 ± 12%) [31]. The discrepancy may be due to different composition ratio and fabricating method used [32]. The excellent swelling property of scaffold facilitated cell seeding and cell adhesion. The number of detached cells in the rinsing solution and medium dropped steeply from 96.0 ± 1.9 × 10^4^ to 49.5 ± 2.4 × 10^3^ within 2 hours. Then it continued to decrease with culture time. Therefore, the scaffold had both excellent biomechanical properties and enlarged surface area for cells adhesion, tissue ingrowths, and nutrients supply [33].

The ligament-bone junction plays a key role in ACL reconstruction. The lack of biological fixation remains the primary cause of graft failure. Due to the complicated structure and various cells, the ligament-bone junction is difficult to successfully reconstruct. In our previous work, the structure of interface was not successfully reconstructed with knitted silk scaffold and MSCs in bone-tunnel. In this study the gene-modified scaffold was used to regenerate the ligament-bone junction.

MSC is known to possess the ability of self-renewal and differentiation into various lineages [34]. In this study MSCs seeded on the hybrid scaffold proliferated robustly and showed good viability. The expression of cartilage-specific markers (collagen II) was upregulated at high levels with the increase of TGF-b3. This is correlated with other reports [35]. The collagen production was also found to increase with culture time.

## 5. Conclusions

This study has successfully developed a gene-modified silk cable-reinforced CHS hybrid scaffold with potential application in reconstructing the cartilage layer of ligament-bone junction. Lentivirus vector was imported by PS coating on scaffold. TGF-*β*3 released by transfected MSCs could induce the chondral differentiation of MSCs. The reinforced silk cables significantly increased the tensile strength of scaffold to meet the mechanical requirements. Future study will focus on introducing multivectors into the hybrid scaffold in order to reconstruct the structure of ligament-bone junction.
